# Risk profiling in the prevention and treatment of chronic wounds using artificial intelligence

**DOI:** 10.1111/iwj.13952

**Published:** 2022-09-21

**Authors:** Karen Cross, Keith Harding

**Affiliations:** ^1^ CEO Mimosa Diagnostics; ^2^ Editor‐in‐Chief, IWJ

For many acute and chronic pathologies, poor individual outcomes and overall statistics reflect the current unsatisfactory state of healthcare.^1^ Severe chronic pathologies such as cardiovascular disorders, diabetes, and cancer are treated after the onset of the disease, frequently at near end stages.^2^, ^3^ Replacing the current paradigm of reactive medical treatment with an innovative approach incorporating predictive, preventive, and personalised medicine (PPPM) is crucial for modern healthcare.^4^

The prevalence and economic burden of wounds are not insignificant, with the United States alone spending over $25 billion annually treating chronic wounds in over 6 million patients. Chronic wounds may result from a range of suboptimal health conditions and severe pathologies (eg, diabetes). Both modifiable and non‐modifiable risk factors make prevention and management highly individual. Individualised patient profiling utilises systems medicine, predictive diagnostics, prevention, and treatment tailored to the individual person, that is, the innovative approaches of PPPM. How is this complex process achievable in wound care?

In this editorial, we are trying to demonstrate how the implementation of complex system thinking may allow us to better address the complex nature of wounds of any aetiology. Some of this started with approaches like the “Wound Bed Paradigm”^5^, ^6^ where the complexity of wound management was thought of in a structured thought system (ie, “complex system thinking”). Other programmes followed, like “Triangle of Wound Assessment”^7^ and MOIST.^8^ This did change the way in which wounds were managed, but it was still a long, tedious journey with regard to helping the development of the clinical area as a speciality.^9^

Artificial intelligence (AI) “is the ability of a machine to perform cognitive tasks to achieve a particular goal based on provided data.”^10^ This new approach is revolutionising and reshaping healthcare systems around the world. The ever‐increasing computational power of AI's highly developed and clinically relevant algorithms, in addition to advanced image processing software, is changing the assessment and diagnosis of patients through medical imaging. This combined power permits the “cognitive” computer to scan billions of pieces of unstructured information, extract the relevant data, and recognise complex patterns with increasing confidence through mass iterative learning,^11^ something that would be impossible for human clinicians to do irrespective of competence and training.

So, decision support systems based on machine learning (ML) have the potential to revolutionise medicine by performing complex tasks that are currently assigned to specialists, improving diagnostic accuracy, increasing efficiency, improving workflow, decreasing costs, and improving treatment choices.^12^, ^13^ Such benefits could be especially helpful in the management of persons with wounds. Technological approaches can enhance clinical performance in diagnostic imaging, interventions, skills training and assessment, digital pathology, and being able to “see under or into the skin.”

Healing prediction is one of the most challenging issues in wound management and is a key component to achieve wound closure.^14^ Most health conditions are complex, but none more so than chronic wounds. The multifactorial complex nature of wounds, in particular those that are chronic, makes prediction by humans difficult (ie, not an exact science). This complexity arises from the complex interaction of many interrelated factors, both wound and patient related. As often stated by those who care for patients with wounds, “it's not about the hole in the patient but rather the whole patient.”^6^ Therefore, this complex web of factors includes other health information outside of the wound itself.^15^, ^16^, ^17^

In today's fast‐moving technologically enhanced world, diagnosis by machines is still in its evolution, although, growing rapidly in many facets of clinical care.^12^, ^18^ Wound care needs to adapt to this changing world to develop highly accurate AI‐based decision‐support applications to improve patient care. This journey has started with the development of wound measurement apps,^19^ treatment decision support apps,^20^ and more recently, wound imaging devices.^21^, ^22^, ^23^ The use of such approaches is changing the way in which persons with wounds are both assessed and managed. From treatment to prevention, this technological approach will change the wound care landscape.

Preventive intervention relies on the identification of regularities or risk profiles, moving from risk factors to risk pattern recognition.^24^ Risk profiling is natural for clinicians but is not an easy task, requiring significant clinical skill and time. The adoption of a complex systems approach^18^, ^25^, ^26^ may push wound care forward in terms of concepts and methods to improve healing prediction, for example. In this sense, moving research from isolated risk factors to “wound” pattern recognition, by means of identification of the complex pattern of interactions among the web of the wound and patient impactors, is necessary.^14^, ^27^, ^28^ A sort of Wound Bed Preparation (WBP) on steroids!

Although difficult, it is feasible to identify and even understand the regularities of a web of impactors using real data and statistical modelling. But this would take time and huge resources to even begin this task. Some of this work has been started over the past two decades, but little progress has been made compared with the potential of AI and ML.^18^

However, there are several challenges in the clinical use of AI‐based applications and interpretation of the results, including data privacy, poorly selected/outdated data, selection bias, and unintentional continuance of historical biases/stereotypes in the data, which can lead to erroneous conclusions.^3^ With an understanding and continued modification of the algorithms behind AI, clinicians can and will adopt this type of approach in the prevention and management of chronic wounds. AI is a transformative technology and has immense potential in wound care.
